# Changes within the coral symbiosis underpin seasonal trophic plasticity in reef corals

**DOI:** 10.1093/ismeco/ycae162

**Published:** 2025-03-14

**Authors:** Emily Chei, Inga Elizabeth Conti-Jerpe, Leonard Pons, David Michael Baker

**Affiliations:** School of Biological Sciences, Swire Institute of Marine Science, The University of Hong Kong, Hong Kong, SAR, China; Science Unit, Lingnan University, Hong Kong, SAR, China; Tropical and Subtropical Research Center, Korea Institute of Ocean Science and Technology, Jeju 63349, Republic of Korea; School of Biological Sciences, Swire Institute of Marine Science, The University of Hong Kong, Hong Kong, SAR, China

**Keywords:** symbiosis, coral, seasonality, trophic strategy, trophic plasticity, stable isotope analysis

## Abstract

Scleractinian corals are mixotrophic organisms that use both autotrophic and heterotrophic pathways to fulfill their metabolic needs. Corals span a spectrum of trophic strategies and vary in their dependence on associated algal symbionts, with certain species capable of increasing heterotrophic feeding to compensate for the loss of autotrophic nutrition. As this ability can improve the likelihood of survival following marine heat waves and environmental disturbance, the continued threat of global and local stressors necessitates the investigation of trophic plasticity to determine coral responses to changing conditions. Here, we examined trophic strategy shifts between wet (high temperature and light) and dry (low temperature and light) seasons for seven genera of scleractinian corals by applying a Bayesian statistical model to determine the isotopic niches of paired coral hosts and their symbionts. Using a novel index (Host Evaluation: Reliance on Symbionts), trophic strategy was evaluated along a continuum of mixotrophy for each season. Three genera exhibited significant trophic shifts and were more heterotrophic in the dry season, likely as a mechanism to compensate for decreased symbiont functioning under lower temperatures and irradiance during these months. The magnitude of trophic plasticity varied across genera, and this pattern was positively correlated with global distribution. Together, our findings substantiate taxonomic differences in nutritional flexibility and provide support for trophic plasticity as a distinguishing trait for understanding coral biogeography.

## Introduction

Symbioses between species are ubiquitous across ecosystems, many of which have evolved into obligate relationships that confer essential benefits to both partners. Among the most common symbioses are those formed with microorganisms, in which hosts and symbionts exchange nutrients and increase their cumulative productivity by accessing a wide range of energy sources [1, 2]. Microbial symbioses expand the metabolic capabilities of the holobiont (the collective assemblage of a host and its symbionts) and increase overall robustness. For example, gut bacteria regulate the health of nearly all vertebrates and invertebrates, [3] and mycorrhizal fungi facilitate essential nutrient uptake in plants [4, 5]. Such associations can broaden a species’ ecological and trophic niche by providing nutritional and energetic resources that are otherwise inaccessible [1, 6, 7]. Studying key symbiotic relationships is necessary to understand the mechanisms underpinning species survival, insight that is vital as human activity drives rapid environmental change.

Among the organisms most vulnerable to environmental change are scleractinian corals that maintain a nutritional symbiosis with unicellular dinoflagellate algae from the family Symbiodiniaceae. This symbiosis has allowed corals to proliferate in oligotrophic oceans [8]; however, anthropogenic impacts such as eutrophication and climate change are rapidly altering the conditions on reefs and disrupting the coral-algal symbiosis [9]. Excess nutrients in the marine environment, particularly nitrogen, can act as a catalyst for rapid symbiont growth [10, 11] and destabilize the coral holobiont by placing a metabolic strain on the host [12]. Symbionts can also behave similarly under higher temperatures, parasitizing the coral host by becoming a greater energetic burden without increasing photosynthate translocation [13]. A breakdown of the host–symbiont relationship can be catastrophic for corals and may result in bleaching, the act of expelling symbiotic algae due to environmental stress (e.g. elevated temperatures) [14].

The ability to modulate reliance on autotrophically derived nutrients is hypothesized to confer stress resistance to corals [15]. Corals are mixotrophic, relying on autotrophic nutrition acquired by their symbionts in the form of photosynthetically fixed carbon, inorganic nitrogen, and phosphorous [16–18], as well as heterotrophic sources of energy such as plankton and dissolved organic nutrients [19]. Corals exhibit interspecific differences in trophic strategy, or the mode of nutrient acquisition, ranging along a continuum of mixotrophy from primarily autotrophic (highly dependent on their symbionts for nutrition) to primarily heterotrophic (limited reliance on symbionts for nutrition) [20]. The amount of resource sharing between hosts and symbionts is positively correlated with bleaching susceptibility [20], providing evidence that heterotrophy is advantageous when the coral-algae symbiosis is strained or disrupted [15, 21].

The ability of a coral species to shift their relative reliance on autotrophy and heterotrophy (trophic plasticity) may be beneficial as an acclimatization response to environmental stress or resource limitation [22–24]. Greater resilience and survival has been observed in species that are able to increase heterotrophy when autotrophic nutrition becomes limited, including bleaching events [15] or periods of high turbidity [25]. The capacity for nutritional flexibility is not equal among corals; it is species-specific and can be influenced by symbiont communities [26, 27] or shaped by environmental conditions [15, 24, 28]. Consequently, trophic plasticity is a fundamental element of a coral’s ecological niche and may underlie a species’ ability to tolerate chronic and acute disturbances, yet most available studies focus on a single species, and direct comparisons of trophic plasticity across multiple species are limited [24, 29–31]. This lack of information restricts understanding of how corals differ in their response to environmental change and limits our ability to predict how coral communities may shift under local and global stressors.

Seasonality presents an opportunity to evaluate coral trophic plasticity as a result of temporal variation in environmental conditions. Many coral reefs experience distinct seasonal differences in temperature and light availability [32]; lower sea surface temperature and radiation during winter months have been correlated with increased symbiont density and pigmentation [33, 34] but decreased calcification [35, 36] and polyp growth [37, 38]. Seasonal fluxes may also play a role in moderating coral resilience. For example, corals in the Caribbean exhibit higher susceptibility to heat stress and bleaching in the summer compared to winter, indicating the potential importance of seasonal effects on a coral’s overall stress response [39]. Investigating trophic plasticity may elucidate the mechanisms driving seasonal acclimatization in corals by discerning changes within the holobiont across different conditions.

Stable isotope analysis (SIA) provides an established method to assess nutritional pathways within the coral holobiont. Carbon (C) and nitrogen (N) stable isotopes reflect an organism’s diet, as δ^13^C is indicative of the carbon source and δ^15^N is enriched with every increase in trophic level [40]. When considering a population, δ^13^C and δ^15^N values are representative of a species’ total resource use and can be used to construct a species’ isotopic niche, a proxy for trophic niche [41]. Applied to coral hosts and symbionts, isotopic niches are useful for evaluating the amount of resource sharing between symbiotic partners by determining the amount of niche overlap between them [20]. This approach has been further refined as a way to quantify trophic strategy by combining overlap parameters into a single metric [42]. Stable isotope tools are valuable for investigating trophic plasticity, and they are increasingly used to determine coral responses during instances of environmental stress [15, 43, 44].

This study examined the trophic plasticity of seven coral genera in situ, the most extensive comparison to date. We evaluated the trophic strategies of each genus in both the wet (summer) and dry (winter) seasons of Hong Kong, which vary considerably in temperature, light, and rainfall [45]. We hypothesized that (1) the capacity for trophic plasticity differs across coral genera, and (2) corals that exhibit seasonal trophic plasticity are more heterotrophic in the dry season to compensate for decreased light availability during the winter months. Metrics derived from carbon and nitrogen stable isotope values were used to examine trophic shifts between seasons and combined with global species distributions to assess the ecological role of trophic plasticity.

## Materials and methods

### Study area and sample collection

**Figure 1:**
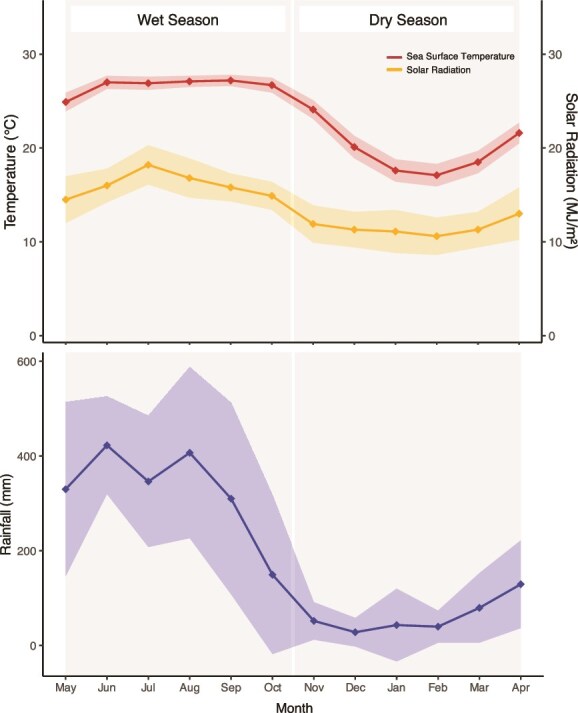
Monthly average values ± SD (shaded areas) grouped by season of annual sea surface temperature, solar radiation, and rainfall in Hong Kong from 2010 to 2020. Data were obtained from the Hong Kong observatory [48].

Hong Kong (22°18’N, 114°10′E) is a subtropical coral region located in the South China Sea. Its climate, driven by monsoons, is separated into two distinct seasons: the wet season, which accounts for 80% of all annual precipitation (May to October), and the dry season (November to April). Monthly mean sea surface temperatures range between 17°C in February and 27°C in August, although annual temperatures can range between 14°C and 32°C [46] (Fig. 1). As a result, coral communities in Hong Kong exist close to the upper and lower limits of their thermal tolerance [47].

From 2013–2015, coral fragments (~5 cm^2^) were haphazardly collected from 52 marine sites throughout Hong Kong for SIA. Up to three colonies of every coral genus present were sampled at each site. Similar morphologies were chosen for sampling, but due to uncertainties with species-specific morphological traits and broad sampling efforts, corals were identified to the genus level. Sites were chosen to encompass most known locations in Hong Kong with coral communities [46]. Using SCUBA, fragments were broken off from the parent colony using a hammer and chisel and placed into individual sampling bags (Whirl-Pak®, Nasco, Pleasant Prairie, WI, USA). Fragments were rinsed twice with deionized water, placed on ice, and transported to the laboratory where they were frozen at −20°C until further processing.

Due to the turnover time of several months needed for coral tissues to reflect changes in isotopic signature [49,50], samples collected from September to November were considered representative of the wet season, and samples collected from April to June were considered representative of the dry season. From the total samples, seven genera (*Acropora*, *Goniopora*, *Montipora*, *Pavona, Platygyra*, *Porites*, and *Oulastrea*) had sufficient replication (*n* ≥ 20) in both seasons for subsequent analyses [51]. These genera represent a broad range of trophic strategies [20].

### Sample processing

Samples (*n* = 442) were processed between 2014 and 2015. Additional samples (*n* = 76), preserved at −20°C from the original sampling efforts, were processed in 2020–2021 to ensure sufficient replication (*n* ≥ 20) of each genus in both seasons to accurately estimate isotopic niche width [51]. For each sample, coral tissue was defrosted and removed from the skeleton using a pressurized airbrush (Single Action Internal Mix Airbrush, Paasche, Kenosha, WI, USA) containing deionized water, and the resulting slurry was homogenized for 30 seconds using a Tissue-Tearor (Model 985 370, BioSpec Products, Inc., Bartlesville, OK, USA).

For samples processed in 2014–2015, the homogenate was centrifuged at 1700 RCF for 5 minutes to separate the host (contained in the supernatant) and symbiont (contained in the pellet) [20]. The host fraction was decanted, and the symbiont pellet was resuspended in 1 ml deionized water and centrifuged at 600 RCF for 5 minutes to produce a clean pellet. Samples in 2020–2021 were processed with a similar protocol that optimized the yield of both host and symbiont fractions using varying speeds depending on the genus [52] (Table S1). The homogenate was centrifuged for 5 minutes to separate host tissue and algal symbionts. The supernatant containing the host fraction was decanted and centrifuged three times for 5 minutes at increasing speeds, with the resulting pellets discarded after each cleaning. Symbionts were resuspended in 1.5 ml deionized water and centrifuged at a low speed for 30 seconds to pellet out any residual skeletal fragments present in the sample. The resulting supernatant was carefully collected and rinsed three times by resuspending symbionts in deionized water and centrifuging for 5 minutes at decreasing speeds to ensure that no host tissue remained. For all samples in both processing periods, the host fraction was examined under a light microscope to confirm the absence of symbiont cells.

Host and symbiont fractions were freeze dried overnight. Host tissue was weighed into tin capsules (1.5 ± 0.1 mg). Symbiont tissue was weighed into silver capsules (1.0 ± 0.1 mg), treated with 5 μl 6 N sequencing-grade HCl to remove any remaining inorganic carbonate, and oven dried overnight at 60°C. All samples were combusted and analyzed for carbon and nitrogen stable isotopes at the University of Hong Kong’s Stable Isotope Laboratory using an elemental analyzer (Eurovector EA3028, Pavia, Italy) coupled to a stable isotope ratio mass spectrometer (Nu Instruments Perspective, Wrexham, UK). Analytical precision was determined using an internal acetanilide standard (iACET; Indiana University, IN, USA) and was better than 0.2‰ for both δ^15^N and δ^13^C.

### Summary statistics and statistical analysis

Isotopic niches were estimated using Stable Isotope Bayesian Ellipses in R (SIBER) [53]. To determine the most probable amount of overlap between host and symbiont groups, Bayesian estimates of two ellipse areas were calculated: (i) the standard ellipse area (SEA_B_), which encompassed 40% of the variation in each group, and (ii) the major ellipse area (MEA_B_) which encompassed 95% of the variation in each group [20, 53]. Models were run with the Markov Chain Monte Carlo parameters of 1000 burn-ins, then 20 000 iterations, with 2 chains, and thins = 10. Relative positions of isotopic niches were assessed by measuring Euclidean distance between host and symbiont ellipse centroids [54].

To visualize niches in isotopic space and calculate additional overlap metrics (see below), maximum likelihood ellipses, encompassing 40% (standard ellipse area, SEA_C_) and 95% (major ellipse area, MEA_C_; 44]) of the data were applied to paired δ^13^C and δ^15^N values and used to determine ellipse area corrected for sample size for both coral host and symbiont. Estimates of the overlapping area of host and symbiont ellipses were produced for both ellipse sizes (40% ellipses = standard ellipse overlap, SEO; 95% ellipses = major ellipse overlap, MEO), where a large percentage of overlap indicated high amounts of resource sharing between symbiotic partners (more autotrophy), and little or no overlap indicated less resource sharing within the symbiosis (more heterotrophy). Standard ellipse areas provided a more conservative estimate of autotrophy due to the lower likelihood of overlap, whereas the larger major ellipse areas provided a more conservative estimate of heterotrophy due to greater likelihood of overlap [42].

Trophic strategy was assessed for each genus in each season using the Host Evaluation: Reliance on Symbionts (HERS) index which combines metrics produced by SIBER into a numerical score ranging from 0 to 1 [42]. HERS incorporates SEO and MEO standardized by (i) host area, estimating the symbiont contribution to total host nutrition, and (ii) symbiont area, estimating the relative amount of symbiont resources assimilated by the host (Equation 1).


(1) \begin{align*} {\mathrm{HERS} = \frac{{\left(\mathrm{SEO}/{\mathrm{SEA}}_{\mathrm{C}}\mathrm{Host}\right)}^{\exp \left(-\mathrm{SEO}/{\mathrm{SEA}}_{\mathrm{C}}\mathrm{Sym}\right)}+{\left(\mathrm{MEO}/{\mathrm{MEA}}_{\mathrm{C}}\mathrm{Host}\right)}^{\exp \left(-\mathrm{MEO}/{\mathrm{MEA}}_{\mathrm{C}}\mathrm{Sym}\right)}}{2}} \end{align*}

A bootstrapping approach was used to calculate HERS scores and test for trophic strategy shifts between seasons [55]. Each genus and season was resampled with replacement from the original data set (*n* = 10 000 replications), and a HERS score was calculated for every iteration to generate a sampling distribution. Trophic strategy was evaluated on a linear scale using mean HERS scores of each genus’s sampling distribution, where 0 represents minimal host and symbiont nutrient sharing or recycling (highly heterotrophic), and 1 represents substantial nutrient sharing or recycling (highly autotrophic). In total, 90% confidence intervals of the distributions were used to test for significant differences between seasons for each genus [56]. The magnitude of trophic plasticity (MP) was quantified for each genus as the absolute value of the difference between wet and dry season mean HERS score (Equation 2).


(2) \begin{align*} {\mathrm{MP} = \mid\left({\mathrm{HERS}}_{\mathrm{Wet}\ \mathrm{Season}}\right)-\left({\mathrm{HERS}}_{\mathrm{Dry}\ \mathrm{Season}}\right)\mid} \end{align*}

To investigate the role that trophic plasticity may play in bounding species distributions, we examined the relationship between the MP of each genus and the geographic distribution of the most common species in Hong Kong within each genus (*Acropora digitifera, Goniopora columna*, *Montipora peltiformis*, *Oulastrea crispata*, *Pavona decussata*, *Platygyra carnosus*, and *Porites lutea*) [57]. The known global ranges were obtained from Corals of the World [58] and represented as the total number of Marine Ecoregions of the World (MEOW) in which each representative species is found [59]. A log-linear model was applied to determine if there was a significant relationship between mean MP and geographic distribution.

## Results

### δ^13^C and δ^15^N values of coral hosts and symbionts

**Figure 2:**
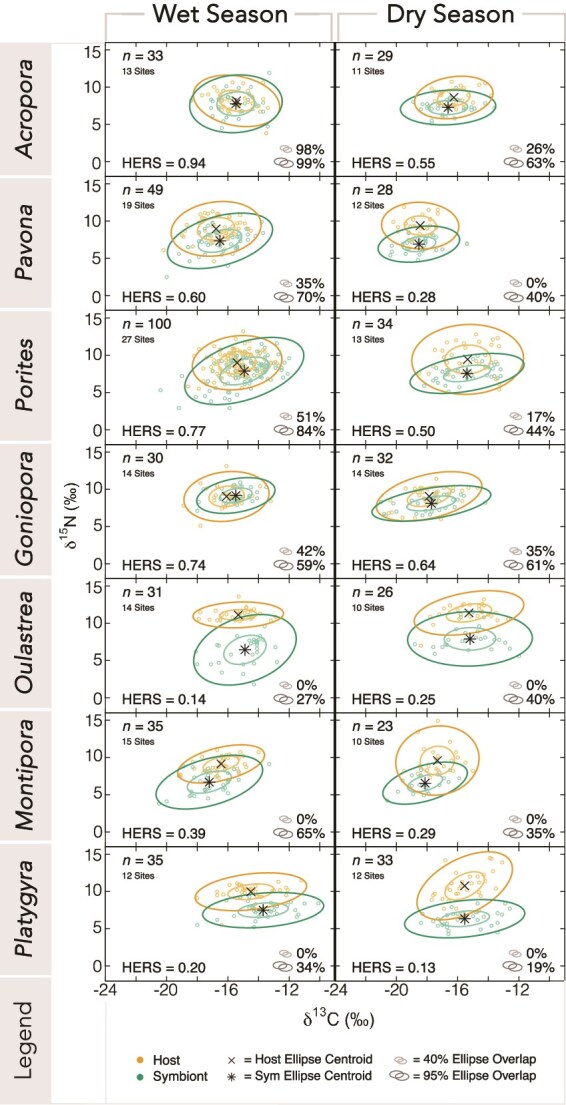
SIBER analyses of paired host and symbiont stable isotope values in both wet and dry seasons, ordered from highest to lowest MP. Ellipse overlap percentages are presented as Bayesian estimates for ellipses containing 40% (SEA_B_; inner ellipse) and 95% (MEA_B_; outer ellipse) of the data, calculated as a proportion of the host area. ellipse centroids are marked by a cross (host) and asterisk (symbiont). HERS scores are evaluated on a linear scale from 0 (highly heterotrophic) to 1 (highly autotrophic). Total number of collection sites are indicated for each genus and season.

δ^13^C values across genera were similar between coral hosts and symbionts, but both had higher values in the wet season relative to the dry season (Fig. 2, Table S2). In the wet season, mean δ^13^C values of coral hosts ranged from −14.4 ± 1.5‰ (*Platygyra*_wet_) to −16.7 ± 1.2‰ (*Pavona*_wet_), while hosts in the dry season ranged from −15.3 ± 1.5‰ (*Porites*_dry_) to −18.4 ± 1.0‰ (*Pavona*_dry_). Symbiont mean δ^13^C values ranged from −13.6 ± 1.6‰ (*Platygyra*_wet_) to −17.3 ± 1.7‰ (*Montipora*_wet_) in the wet season and − 15.3 ± 1.5‰ (*Porites*_dry_) to −18.1 ± 1.1‰ (*Montipora*_dry_) in the dry season.

The δ^15^N values of coral hosts were consistently higher than symbionts across seasons (Table S2). Mean δ^15^N values ranged from 8.1 ± 1.2‰ (*Acropora*_wet_) to 10.9 ± 0.9‰ (*Oulastrea*_wet_) for hosts in the wet season. In the dry season, mean δ^15^N values ranged from 8.6 ± 1.2‰ (*Acropora*_dry_) to 10.7 ± 1.9‰ (*Platygyra*_dry_), with *Acropora* exhibiting the lowest mean δ^15^N values for both seasons. For symbionts, mean δ^15^N values ranged from 5.8 ± 2.4‰ (*Oulastrea*_wet_) to 9.1 ± 0.9‰ (*Goniopora*_wet_) in the wet season and 6.5 ± 1.1‰ (*Montipora*_dry_) to 8.1 ± 0.9‰ (*Goniopora*_dry_) in the dry season.

### Stable Isotope Bayesian Ellipses in R percent overlap and centroid distance

Overlap of host and symbiont ellipses as a proportion of host area varied greatly between genera and season. For the Bayesian estimates of standard ellipse area (SEA_B_), overlap ranged from 0% to 98%, and overlap estimates for major ellipse area (MEA_B_) ranged from 19% to 99% (Fig. 2, Table S3, Table S4). The greatest amount of overlap was observed in the wet season for *Acropora*_wet_ with almost complete overlap of host and symbiont isotope niches (SEA_B_ = 98%, MEA_B_ = 99%) and *Porites*_wet_ (SEA_B_ = 51%, MEA_B_ = 84%), while overlap in the dry season was greatest for *Acropora*_dry_ (SEA_B_ = 26%, MEA_B_ = 63%) and *Goniopora*_dry_ (SEA_B_ = 35%, MEA_B_ = 61%). Among all genera, *Oulastrea* (*Oulastrea*_wet_ SEA_B_ = 0%, MEA_B_ = 27%; *Oulastrea*_dry_ SEA_B_ = 0%, MEA_B_ = 40%) and *Platygyra* (*Platygyra*_wet_ SEA_B_ = 0%, MEA_B_ = 34%; *Platygyra*_dry_ SEA_B_ = 0%, MEA_B_ = 19%) had the lowest amount of overlap across seasons.

*Acropora*, *Pavona*, *Porites*, *Montipora*, and *Platygyra* exhibited less overlap of both SEA_B_ and MEA_B_ in the dry season than the wet season. In contrast, *Oulastrea* had less overlap during the wet season than the dry season, whereas *Goniopora* had greater SEA_B_ overlap in the wet season than the dry season but exhibited the opposite pattern for MEA_B_ (*Goniopora*_wet_ SEA_B_ = 42%, MEA_B_ = 59%; *Goniopora*_dry_ SEA_B_ = 35%, MEA_B_ = 61%). Distance between host and symbiont ellipse centroids ranged from the lowest values of 0.4‰ (*Acropora*_wet_) and 0.6‰ (*Goniopora*_wet_) to the highest values of 4.4‰ (*Platygyra*_dry_) and 4.7‰ (*Oulastrea*_wet_).

### Host Evaluation: Reliance on Symbionts scores and trophic plasticity

**Figure 3:**
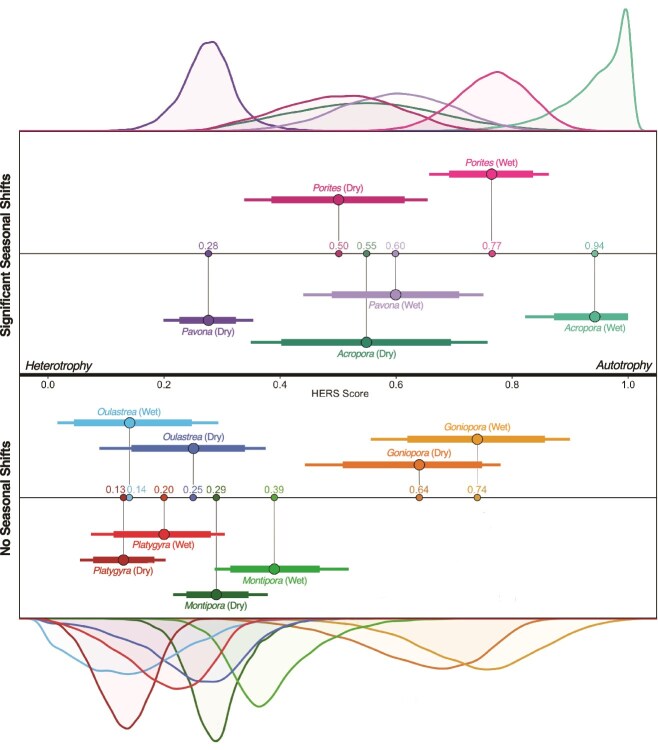
Bootstrapped HERS scores and density plots (*n* = 10 000 replications) for three coral genera with significant trophic strategy shifts (*Acropora*, *Pavona*, *and Porites*) and four genera without significant trophic strategy shifts (*Oulastrea*, *Goniopora*, *Montipora*, and *Platygyra*) between the wet and dry seasons. Dots and the associated value represent the mean, and error bars represent 75% and 90% confidence intervals.

**Figure 4:**
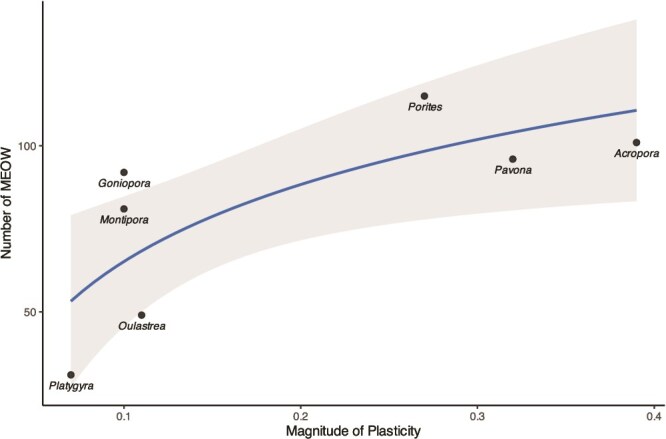
Modeled relationship between mean MP and number of MEOW of all coral genera, represented by the most common species found in Hong Kong (adjusted R^2^ = 0.49, *P* = .04). Line represents predicted values based on the linear regression model of log-transformed data. Shaded area represents a 95% confidence interval.

Mean HERS scores of all genera spanned nearly the entire range of scores from 0.94 (*Acropora*_wet_, 90% CI: 0.82, 1.00) to 0.14 (*Oulastrea*_wet_, 90% CI: 0.01, 0.29) and 0.13 (*Platygyra*_dry,_ 90% CI: 0.05, 0.20; Fig. 3). *Acropora* displayed the greatest magnitude of plasticity (MP = 0.39), followed by *Pavona* (MP = 0.32) and *Porites* (MP = 0.27). Bootstrapped 90% confidence limits indicated significant trophic strategy shifts between seasons, with lower mean HERS scores in the dry season, for *Acropora, Pavona,* and *Porites* (Fig. 3). *Platygyra* (MP = 0.07), *Montipora* (MP = 0.10), *Oulastrea* (MP = 0.11), and *Goniopora* (MP = 0.10) were the least plastic genera and did not exhibit significant shifts between seasons (Fig. 4). A log-linear regression showed a significant relationship between mean MP and number of MEOW (R^2^ = 0.49, *P* = .04) with greater plasticity correlating with greater distribution area across ecoregions (Fig. 4).

## Discussion

### Seasonal trophic plasticity varies across trophic strategy

Trophic plasticity has been identified as a mechanism through which corals tolerate a wider range of conditions, including those that induce physiological stress. Our results indicate that the capacity for trophic plasticity varies across corals, with some species maintaining a relatively high reliance on heterotrophy across seasons (*Montipora*, *Platygyra*, and *Oulastrea*) while others shifted from a strong reliance on autotrophy in the wet season (May to October) to an increased reliance on heterotrophy in the dry season (November to April; *Acropora*, *Pavona*, and *Porites*; Fig. 3). Previous studies have similarly observed that some corals are able to shift trophic strategy across seasons [29, 60] and depths [24, 61], as well as in response to bleaching [15] and turbidity [25]. Seasonal trophic shifts have been found in corals from both temperate [60] and tropical [29] environments, with plastic species generally exhibiting more heterotrophy in the winter and wet season, respectively. These patterns have been explained through two different mechanisms: in temperate corals, reduced light during the winter months was hypothesized to limit photosynthesis of associated Symbiodiniaceae which the coral compensated for through increased heterotrophy [60]. In tropical environments, higher amounts of precipitation in the wet season increase turbidity and reduce the light available to corals, similarly limiting photosynthesis, although the response is species-specific [29]. Our results are congruent with the trophic shifts found in temperate corals, supporting seasonal light intensity and temperature as the drivers of trophic strategy shifts in Hong Kong rather than changes in turbidity caused by runoff in the wet vs. dry season.

Limitation of algal symbiont photosynthesis in the dry season is further supported by the fact that significant trophic shifts only occurred in three highly autotrophic genera: *Acropora*, *Pavona*, and *Porites* (Fig. 3). All three of these genera exhibited high (>0.5) HERS scores in the summer wet season, indicating more reliance on autotrophy, and lower HERS scores in the winter dry season, indicating an increased reliance on heterotrophy. Changes in symbiont communities across ecological gradients can affect coral trophic status due to hosting Symbiodiniaceae species with varying autotrophic capabilities [62], but Hong Kong corals exhibit high fidelity to symbiont type regardless of depth, space, seasons, and after bleaching disturbance [63–65]. Instead, as found in temperate corals [60], we posit that these shifts were driven primarily by climatic factors, such as solar radiation and temperature, which limit photobiont productivity in the dry season (November to April). Light limiting conditions have been found to decrease photosynthate production and translocation [66]. Similarly, sustained low temperatures have been shown to decrease the photosynthetic efficiency of corals [66, 67] while sharp declines in temperature can induce cold stress that result in reduced symbiont density [68]. Hong Kong is on the edge of the range of reef-building corals, with temperatures optimal for productivity occurring ~50% of the year and several Hong Kong species—including *Acropora samoensis*, *Montipora caliculata*, *Oulastrea crispata*, and *Porites lobata*—exhibiting declines in productivity measured through photosynthesis and respiration (P:R) ratios at temperatures below 21°C–23°C [69].

We hypothesize that the relatively harsh winter conditions in Hong Kong limit the photosynthetic output of algal symbionts in the dry season, reducing or eliminating autotrophy as a viable source of nutrition for their coral hosts. This diminished autotrophy drives corals to increase dependence on heterotrophic sources of nutrition [36, 70], resulting in the separation of host and symbiont isotopic niches (Fig. 2). In contrast, these corals exhibit considerable niche overlap during the wet season when temperature and light intensity are elevated, reflecting host dependence on autotrophic contributions. Our data do not support changes in lipid reserves as a mechanism driving seasonal shifts in isotope values. Storage lipids are typically most depleted during the winter and highest in the summer, peaking with lipid stockpiling for gametogenesis and spawning [71, 72]. However, carbon changes do not reflect the signals expected from the seasonal catabolism or synthesis of ^12^C-rich lipids [73]. Rather, lower overall δ^13^C values occur during the dry season (Table S2), which is consistent with patterns of light-mediated fractionation, where reduced rates of photosynthesis lead to carbon depletion due to preferential assimilation of ^12^C [74, 75]. Significant seasonal shifts for three genera with high HERS scores therefore suggest that trophic plasticity is a mechanism that more autotrophic corals employ to tolerate seasonality. Our results provide evidence that trophic plasticity acts as an acclimatization strategy for autotrophic coral genera, while others invest heavily in heterotrophy despite seasonal changes.

The corals that exhibited the least trophic plasticity were also generally the most heterotrophic. Of the four genera that did not experience significant trophic strategy shifts between seasons, three had the lowest overall HERS scores (*Montipora*, *Platygyra*, and *Oulastrea*; Fig. 3). Autotrophy is often regarded as the basis of coral nutrition due to its ability to meet >100% of metabolic demand [76]; however, our results indicate that a consistent reliance on heterotrophy throughout the year is an advantageous strategy for certain genera. Previous work has demonstrated that while photosynthetically fixed carbon is quickly respired by the host, heterotrophic carbon is incorporated into lipids, proteins, and nucleic acids [77]. Additionally, nutrients from feeding can be used for multiple physiological functions under different conditions. For example, heterotrophic carbon is used primarily for skeletal growth when light and photosynthesis are restricted, but under high light availability, heterotrophic nutrients are preferentially used to support tissue growth [77, 78]. The versatility of heterotrophic nutrition may be why these corals incorporate it into their diet across seasons and use it to maintain a large portion of their energy budget [31, 79] as long as particulate food remains abundant [80]. It is important to note that these corals maintain some overlap between host and symbiont niches, indicating that they receive autotrophic inputs from Symbiodiniaceae [81] and thus reflect a consistent mixotrophic nutritional mode with a relatively greater input of heterotrophy than other corals. The exception to this pattern was *Goniopora*, a genus that was highly autotrophic but also did not demonstrate significant seasonal shifts. This could be explained by *Goniopora*’s long, extended polyps with increased surface area for autotrophy, an attribute that may allow for continued photosynthesis across varied seasonal conditions, including high sedimentation and low light [82, 83].

The capacity for trophic plasticity of the genera examined in this study contradicted some previous findings. For example, Nahon *et al.* (2013) found tha*t Porites rus*, *Acropora cytherea*, and *Acropora hyacinthus* exhibited relatively static trophic strategies across sites and seasons [29], whereas our data indicated *Porites* and *Acropora* were two of the most plastic genera in Hong Kong. Nahon *et al.* also determined that *Pavona cactus* and *Montipora tuberculosa* had high trophic plasticity, yet only *Pavona* showed a high MP in this study. Further, some species of *Porites* have been found to lack trophic plasticity when faced with heat stress or varying turbidity [15, 25, 29], though others have shown higher rates of feeding with increasing depth [28]. Conflicting reports at the genus level demonstrate that capacity for trophic plasticity is likely a species-specific trait, particularly as many existing studies were conducted using species absent from Hong Kong [57]. Here, species identification through visual or molecular approaches was not possible due to taxonomic uncertainties and insufficient sampling material, but future studies should prioritize classifying individuals to the species level for more accurate comparisons.

The corals included in this study may also be adapted to an environment characterized by seasonal change. Coral communities of the subtropics are exposed to a greater magnitude and frequency of fluctuating environmental conditions than tropical corals [84], thus the trophic strategies we observed could reflect adaptations for tolerating temperature and light availability close to species’ limits. This may explain incongruencies between our findings and those from more stable tropical environments [60]. Additionally, trophic plasticity may be conditional on the nature of the stressor. *Montipora capitata* has been found to increase heterotrophy under heat stress in laboratory conditions [15, 27], though this pattern was not observed for *Montipora* between wet and dry seasons (Fig. 3) or in naturally bleached colonies of *M. capitata* [85]. The lack of seasonal plasticity may not necessarily indicate an inability to shift trophic strategies when other perturbations, such as warming, occur, but more work is needed to delineate the effects of various stressors on coral trophic plasticity.

### The physiological drivers of trophic plasticity

The patterns observed in the overlap of coral and algal isotopic niches indicate changes in the nutrition of *Acropora*, *Pavona*, and *Porites* across seasons*,* with seasonal differences between ellipses driven largely by nitrogen (Fig. 2). While regions of Hong Kong experience baseline δ^15^N shifts between seasons owing to land-based pollution from river discharge [86], this signal would be equally reflected in the nitrogen values of both symbiotic partners unless there was also a concurrent change in the amount of nutrient sharing, further emphasizing a trophic shift in these corals. However, shifts between heterotrophy and autotrophy, as determined by isotope values, are relative to one another rather than due to differences in the absolute contributions of each nutritional source. As a result, the seasonal shifts observed in these three genera could be explained either through an increase in heterotrophy in the dry season or consistent heterotrophy across seasons coupled with a decrease or disruption in nutrient recycling within the holobiont.

Due to reduced symbiont productivity in the dry season, increased heterotrophy may be used to supplement the energetic needs of *Acropora*, *Pavona*, and *Porites.* Heterotrophic organisms undergo trophic fractionation and preferentially excrete light nitrogen (^14^N) in waste products, resulting in an elevated δ^15^N signature in animal tissue relative to the food source [40]. The observed δ^15^N enrichment of host tissue, and subsequent dry season separation of isotopic niches, can therefore be explained by increased feeding by *Acropora*, *Pavona*, and *Porites* compared to the wet season [20, 24]. Similar to trophic plasticity exhibited in corals under heat stress and high turbidity [15, 25], heterotrophy can compensate for reduced photosynthesis, moderating stress from cold temperatures and lower irradiance that corals experience during the dry season. Indeed, similar observations have been made in temperate regions experiencing seasonal temperature fluctuations, where heterotrophy is an important carbon source and symbionts have shown greater reliance on host-derived nutrients during the winter months than in the summer [15, 25, 60].

The heterotrophic signal exhibited during the dry season could also be a result of diminished nutrient recycling*.* In corals, Symbiodiniaceae recycle host nitrogenous waste by synthesizing amino acids from excreted ammonium and translocating these biomolecules back to the host [87]. Given that animals preferentially excrete ^14^N [40], nitrogen recycling effectively reduces trophic fractionation by conserving light nitrogen within the holobiont [88], resulting in isotopic niche overlap. Amino acid synthesis is dependent on the availability of photosynthetic carbon [87], thus impaired symbiont functioning in the dry season may disrupt the recycling of isotopically light nitrogenous waste. Reduced niche overlap and lower HERS scores in the dry season may therefore result from impaired symbiont functioning that eliminates nutrient recycling rather than an uptick in heterotrophy.

Regardless of which mechanism is at play, both increased heterotrophy or reduced recycling involve a relative reduction in the contribution of nutrient sources derived from the symbiotic algae to the coral nutritional budget. Stable isotope data alone are unable to quantify absolute contributions of heterotrophy and nutrient recycling in corals. Future studies can employ isotope tracer experiments and quantification of particle capture to directly measure the assimilation of these sources in species that span the HERS spectrum to “ground-truth” this metric. Despite these uncertainties, the decreased overlap and resource sharing between symbiotic partners demonstrate that plastic hosts are less reliant on autotrophic contributions and more dependent on heterotrophic resources during the dry season, while static hosts maintain a higher relative reliance on heterotrophy throughout the year.

### Trophic plasticity and global distribution

When considering global distribution, we found a significant relationship between trophic plasticity (MP) and the number of ecoregions a species inhabits (Fig. 4). Corals that encompassed the greatest number of marine ecoregions were genera with a higher MP (*Acropora*, *Pavona*, and *Porites)*, whereas corals with lower MP spanned the fewest ecoregions and had the smallest distribution (*Montipora*, *Platygyra*, and *Oulastrea*; Fig. 4). Although many abiotic and biotic factors limit coral distribution (e.g. environmental and climatic factors such as sea surface temperatures, mode of reproduction, larval dispersion, symbiont specificity, competition, and predation) [89–91], the significant relationship between the magnitude of plasticity and number of ecoregions inhabited demonstrates that trophic plasticity is an important trait that influences coral species distribution.

The ability to switch between high reliance on autotrophy and a more mixotrophic strategy may enable corals with a high MP, such as *Acropora*, to be successful across a multitude of ecoregions. Autotrophic capabilities aid *Acropora*’s dominance as reef-builders in ideal habitats: through prioritizing growth rates and productivity in oligotrophic environments [92, 93], *Acropora* are able to outcompete other species and exhibit the highest rates of growth and calcification compared to other genera [94]. Trophic plasticity may maximize the number of ecoregions autotrophic corals inhabit by allowing them to prevail in conditions conducive to photosynthesis (e.g. clear water, high light availability) while persisting in less favorable environments (e.g. high turbidity) [95]. Significant seasonal trophic strategy shifts suggest that trophic plasticity facilitates *Acropora*’s ability to tolerate variable climatic conditions, possibly contributing to its widespread distribution and persistence even at higher latitudes (Fig. 4) [96–98]. Similarly, trophic plasticity may support the extensive distribution of corals like *Pavona* and *Porites* by providing the flexibility to exist across ecoregions, although these genera do not demonstrate the same historical dominance on reefs as branching corals like *Acropora* [99, 100]*.* This could be attributed to slower growth rates associated with plating and massive morphologies [93, 94] and may be indicative of a moderately competitive survival strategy suited for varied environments [101].

*Platygyra*, *Oulastrea*, and *Montipora*, however, were among the genera with the lowest MP and had constricted ranges that covered the fewest ecoregions. As evidenced by low HERS scores across both seasons, these genera maintain a relatively high reliance on heterotrophy and are likely outcompeted by more autotrophic species when light is abundant, but they may be more resilient in conditions of high turbidity or low light that are preferential toward heterotrophic corals [19, 102]. For example, *Platygyra* and *Oulastrea* had the lowest distribution and spanned the fewest ecoregions (Table 1) but are categorized as highly resilient to environmental stressors: *Platygyra* is a massive, stress-tolerant coral that is consistently heterotrophic [103], and *Oulastrea* often inhabits marginal environments [104] and is one of the few species capable of existing beyond the biological and geographical boundaries that inhibit most calcifying corals [105]. Our results indicate that these corals rely primarily on heterotrophy, allowing them to survive in habitats where symbiont photosynthesis is consistently reduced [19]. *Platygyra* and *Oulastrea* may represent corals that are specialized for stress-tolerance [101], but due to a lower capacity to exploit autotrophic pathways, they are likely less competitive in favorable environments and span fewer ecoregions than corals with greater trophic plasticity.

**Table 1:** Distance between host and symbiont centroids (centroid distance, ‰). Mean HERS and 90% confidence intervals calculated from bootstrapped samples of each genus and season, with scores ranging from 0 (highly heterotrophic) to 1 (highly heterotrophic). Mean MP was determined from bootstrapped absolute values of the difference in seasonal HERS scores for each genus. Geographic distribution is represented by the number of MEOW spanned by the representative species of each genus.

Genus	Season	*n*	Centroid distance (‰)	Mean HERS	90% CI	MP	Number of MEOW
*Acropora*	Wet	100	0.40	0.94	0.82–1.00	0.39	101
	Dry	34	1.39	0.55	0.34–0.75		
*Goniopora*	Wet	31	0.62	0.74	0.55–0.89	0.10	92
	Dry	26	0.96	0.64	0.44–0.78		
*Montipora*	Wet	30	2.57	0.39	0.28–0.51	0.10	81
	Dry	32	3.14	0.29	0.21–0.37		
*Oulastrea*	Wet	49	4.70	0.14	0.01–0.29	0.11	49
	Dry	28	3.49	0.25	0.08–0.37		
*Pavona*	Wet	35	1.63	0.60	0.43–0.75	0.32	96
	Dry	33	2.48	0.28	0.19–0.35		
*Platygyra*	Wet	33	2.59	0.20	0.07–0.30	0.07	31
	Dry	29	4.42	0.13	0.05–0.20		
*Porites*	Wet	35	1.21	0.77	0.65–0.86	0.27	115
	Dry	23	1.88	0.50	0.33–0.65		

Overall, the close relationship between MP and HERS scores makes it difficult to effectively disentangle the underlying traits driving biogeography. Rather, we propose that a coral’s trophic strategy influences its capacity for trophic plasticity as demonstrated by differences in seasonal shifts between highly autotrophic and heterotrophic genera. Wang *et al.* (2024) presented similar findings in which trophic plasticity was positively correlated with spatial distribution, and the narrowest distribution was attributed to the most heterotrophic species with the lowest trophic flexibility [106]. This connection suggests that both aspects of a coral’s nutritional mode should be considered when evaluating distribution, though resolving intra-genus differences is required to fully understand these effects given inconsistent reports of trophic plasticity at the species level.

### Conclusions and implications

This study is among the first to assess nutritional flexibility across an extensive range of coral genera [24, 44], and we have applied a recent method in a new way to quantify trophic plasticity in symbioses with diverse nutrient sources. We present evidence of variable trophic plasticity across corals, revealing distinct strategies to tolerate seasonal climatic changes. Corals are traditionally viewed as predominant autotrophs, thus trophic shifts have been framed as the ability or inability to “upregulate” heterotrophy in corals that predominantly rely on their algal symbionts for nutrition. Our results, however, suggest that autotrophic corals typically exhibit plasticity whereas predominant heterotrophs maintain a consistent trophic strategy across seasons. When examined more broadly, we suggest that trophic strategy and plasticity can be used to explain patterns of species survival and coral distribution across distinct environments.

Variation among the seven genera highlight taxonomic differences in coral trophic ecology and may reflect adaptations to fluctuating environmental conditions of the subtropics [84]. With climate change exacerbating the incidence of thermal stress, understanding these mechanisms has increasing relevance as higher latitudes become possible refugia for topical corals [107]. Lower irradiance and greater temperature fluctuations in the subtropics, particularly in the winter, remain major factors limiting coral range expansions to higher latitudes [108]. Insight into seasonal acclimatization processes may have important implications for predicting the future success of coral species in the Anthropocene. The novel metrics described in this study can be applied to additional species across both spatial and temporal scales, and future work should investigate the physiological mechanisms that drive nutritional exchanges within the coral symbiosis.

## Supplementary Material

Supplemental_Tables_Cheietal_2024_ycae162

Supplemental_Script1_Cheietal2024_SIBER_ycae162

Supplemental_Script2_Cheietal2024_HERSBootstrap_ycae162

Dataset_Cheietal_2024_ycae162

## Data Availability

The datasets generated and analyzed during this study are publicly available through referenced databases or are included in this article as supplementary data files.
