# Supplementary material for: Changes within the coral symbiosis underpin seasonal trophic plasticity in reef corals
Source: ISME Commun. 2025 Mar 14;5(1):ycae162. doi: 10.1093/ismeco/ycae162 (PMC11931219; doi:10.1093/ismeco/ycae162)
Supplement: Supplemental_Tables_Cheietal_2024_ycae162 [file supplemental_tables_cheietal_2024_ycae162.pdf]

Supplementary Materials for:

Changes within the coral symbiosis underpin seasonal trophic plasticity in reef corals

Emily Chei[1], Inga Elizabeth Conti-Jerpe[2], Leonard Pons[3], David Michael Baker[1*]

[1] School of Biological Sciences, Swire Institute of Marine Science, The University of Hong Kong, Hong Kong, SAR, China

[2] Science Unit, Lingnan University, Hong Kong, SAR, China

[3] Tropical & Subtropical Research Center, Korea Institute of Ocean Science and Technology, Jeju, Republic of Korea

Corresponding Author: David Michael Baker (dmbaker@hku.hk)

This PDF file includes:

Tables S1 to S4

Supplementary Table 1. Centrifugation speeds for separation of coral host and symbiont fractions. Protocol adapted from Kim et al. (2021).

Genus	Initial Centrifugation (RCF)	Type	1st Centrifugation (RCF)	2nd Centrifugation (RCF)	3rd Centrifugation (RCF)
Acropora *Porites*	300	Host	200	300	450
		Symbiont	200	150	100
Goniopora	300	Host	200	250	350
		Symbiont	200	150	100
Montipora *Pavona*	350	Host	200	300	450
		Symbiont	300	200	150
Oulastrea	400	Host	200	300	450
		Symbiont	300	250	200
Platygyra	800	Host	450	650	800
		Symbiont	650	450	350

Supplementary Table 2. Mean ± SD of $\delta^{13}C$ and $\delta^{15}N$ values for host and symbiont fractions of seven coral genera across wet and dry seasons.

		Wet Season		Dry Season	
**Genus**	**Type**	**$\delta^{13}C$ (‰)**	**$\delta^{15}N$ (‰)**	**$\delta^{13}C$ (‰)**	**$\delta^{15}N$ (‰)**
Acropora	Host	-15.4±1.1	8.1±1.2	-16.2±1.0	8.6±1.2
	Symbiont	-15.4±1.3	7.6±1.7	-16.6±1.3	7.2±0.9
Goniopora	Host	-16.0±1.1	9.0±1.4	-17.8±1.4	9.0±1.3
	Symbiont	-15.4±1.0	9.1±0.9	-17.7±1.6	8.1±0.9
Montipora	Host	-16.4±1.2	9.1±1.1	-17.3±1.1	9.5±1.9
	Symbiont	-17.3±1.7	6.6±1.5	-18.1±1.1	6.5±1.1
Oulastrea	Host	-15.4±1.2	10.9±0.9	-15.6±1.5	10.7±1.6
	Symbiont	-15.1±1.4	5.8±2.4	-15.5±1.7	7.0±2.4
Pavona	Host	-16.7±1.2	8.9±1.5	-18.4±1.0	9.3±1.3
	Symbiont	-16.5±1.4	7.3±1.5	-18.5±1.1	6.9±1.0
Platygyra	Host	-14.4±1.5	9.9±1.0	-15.5±1.3	10.7±1.9
	Symbiont	-13.6±1.6	7.4±0.9	-15.5±1.6	6.3±1.0
Porites	Host	-15.4±1.2	9.0±1.5	-15.3±1.5	9.4±1.9
	Symbiont	-14.9±1.6	7.8±1.8	-15.3±1.5	7.5±1.1

Supplementary Table 3. Bayesian standard ellipse areas (SEA_B) and overlap metrics for seven coral genera in the wet and dry seasons. Posterior distributions of fitted ellipses containing 40% of the data was used to estimate the area for hosts, symbionts, and the overlap between paired ellipses of each genus and season. Overlap was estimated from the distributions as a proportion of host SEA_B and symbiont SEA_B.

Genus	Season	Host SEA_B (‰^{2})	Symbiont SEA_B (‰^{2})	Overlap Area (‰^{2})	Host SEA_B Proportion Overlap	Symbiont SEA_B Proportion Overlap
Acropora	Wet	3.98	6.79	4.00	0.98	0.46
	Dry	3.70	3.59	0.78	0.26	0.23
Goniopora	Wet	4.74	2.97	2.49	0.42	0.74
	Dry	5.27	4.13	2.14	0.35	0.44
Montipora	Wet	3.33	5.83	0.00	0.00	0.00
	Dry	6.32	3.54	0.00	0.00	0.00
Oulastrea	Wet	2.64	7.70	0.00	0.00	0.00
	Dry	5.08	7.58	0.00	0.00	0.00
Pavona	Wet	5.56	5.91	1.73	0.35	0.28
	Dry	3.99	3.23	0.00	0.00	0.00
Platygyra	Wet	4.66	4.69	0.00	0.00	0.00
	Dry	6.50	4.93	0.00	0.00	0.00
Porites	Wet	5.61	8.23	2.82	0.51	0.36
	Dry	8.81	4.61	1.87	0.17	0.36

Supplementary Table 4. Major ellipse areas (MEA_B) and overlap metrics of seven coral genera in the wet and dry seasons. Bayesian distributions were used to determine the posterior estimate of area for hosts, symbionts, and the overlap between paired hosts and symbionts using ellipses containing 95% of the data. Overlap was estimated from the distributions as a proportion of host MEA_B and symbiont MEA_B.

Genus	Season	Host MEA_B (‰^{2})	Symbiont MEA_B (‰^{2})	Overlap Area (‰^{2})	Host MEA_B Proportion Overlap	Symbiont MEA_B Proportion Overlap
Acropora	Wet	25.75	42.68	24.45	0.99	0.55
	Dry	21.94	21.68	14.48	0.63	0.61
Goniopora	Wet	30.30	18.27	17.04	0.59	0.99
	Dry	33.30	26.56	20.95	0.61	0.81
Montipora	Wet	20.95	35.81	13.47	0.65	0.37
	Dry	38.70	21.76	13.81	0.35	0.62
Oulastrea	Wet	16.62	47.99	4.49	0.27	0.09
	Dry	29.91	45.93	12.19	0.40	0.24
Pavona	Wet	33.44	34.23	22.62	0.70	0.64
	Dry	24.39	19.05	9.96	0.40	0.49
Platygyra	Wet	28.72	28.99	9.91	0.34	0.49
	Dry	40.84	31.21	8.72	0.19	0.29
Porites	Wet	33.93	48.94	28.47	0.84	0.57
	Dry	54.39	26.40	26.49	0.44	0.88
